# In-person cognitive behavioural therapy vs. usual care after surgical management of extremity fractures: an unsuccessful feasibility trial

**DOI:** 10.1186/s40814-023-01430-y

**Published:** 2024-01-06

**Authors:** Kyle Gouveia, Sheila Sprague, Jodi Gallant, Gina Del Fabbro, Jordan Leonard, Sofia Bzovsky, Paula McKay, Jason W. Busse, on behalf of the COPE Investigators, Mohit Bhandari, Gerard Slobogean, Lehana Thabane, Randi E. McCabe, Emil H. Schemitsch, Gordon H. Guyatt, PJ Devereaux, I. Leah Gitajn, Matilda Nowakowski, Eleni Hapidou, Delia Chiaramonte, Henrick Kehlet, James Khan, Aresh Sepehri, Natalie Fleming, Christy Shibu, Diane Heels-Ansdell, Brad A. Petrisor, Dale Williams, Bill Ristevski, Jamal Al-Asiri, Herman Johal, Matthew Denkers, Kris Rajaratnam, Sarah MacRae, Kaitlyn Pusztai, Sara Renaud, Nicki Johal, Steven Papp, Karl-Andre Lalonde, Bradley Meulenkamp, Allan Liew, Manisha Mistry, Braden Gammon, Wade Gofton, Geoffrey Wilkin, Melanie Dodd-Moher, David Puskas, Travis Marion, Tina Lefrancois, Jubin Payandeh, Claude Cullinan, Tracy Wilson, Kurt Droll, Michael Riediger, Rabail Siddiqui, Shalyn Littlefield, Simrun Chahal, Paige Wagar, Prism S. Schneider, Tosin Ogunleye, Tanya Cherppukaran, Karin Lienhard, Nicholas Smith, Sarah Anthony, Krista Butt, LaShann Selby, Murali Kovvur, Joshua Lawrence, Skyler Sampson, Kristin Turner, Todd Jaeblon, Haley K. Demyanovich, Sneh Talwar, Caroline Benzel, Theresa Chockbengboun, Devin Mullin, Paul J. Appleton, John J. Wixted, Edward K. Rodriguez, Michael F. McTague, Katiri Wagner, Kristina Brackpool, Kate Hegermiller, Nhi Nguyen, Roman M. Natoli, Courteney Fentz, Maricela Diaz, Jill Niceley, Tammy Garrett, Kyle J. Jeray, Thomas M. Schaller, Michael S. Sridhar, John D. Adams, Richard W. Gurich, Stephanie L. Tanner, Kyle Adams, Michelle Donohue, Emily Bray, Calleigh Brignull, Harper Sprouse, Christina Tieszer, Trevor Stone, Darius Viskontas, Mauri Zomar

**Affiliations:** 1 Division of Orthopaedic Surgery, Department of Surgery, McMaster University, 293 Wellington St. N, Suite 110, Hamilton, ON L8L 8E7 Canada; 2 Department of Health Research Methods, Evidence, and Impact, McMaster University, Hamilton, Ontario Canada; 3 Department of Anesthesia, Michael G. DeGroote School of Medicine, McMaster University, Hamilton, Ontario Canada

**Keywords:** Extremity fractures, Persistent post-surgical pain, Cognitive behavioral therapy

## Abstract

**Background:**

Extremity fractures are common, and most are managed operatively; however, despite successful reduction, up to half of patients report persistent post-surgical pain. Furthermore, psychological factors such as stress, distress, anxiety, depression, catastrophizing, and fear-avoidance behaviors have been associated with the development of chronic pain. The purpose of this pilot study was to examine the feasibility of a randomized controlled trial to determine the effect of in-person cognitive behavioral therapy (CBT) vs. usual care on persistent post-surgical pain among patients with a surgically managed extremity fracture.

**Methods:**

Eligible patients were randomized to either in-person CBT or usual care. We used four criteria to judge the composite measure of feasibility: 1) successful implementation of CBT at each clinical site, 2) 40 patients recruited within 6 months, 3) treatment compliance in a minimum 36 of 40 participants (90%), and 4) 32 of 40 participants (80%) achieving follow-up at one year. The primary clinical outcome was persistent post-surgical pain at one year after surgery.

**Results:**

Only two of the four participating sites were able to implement the CBT regimen due to difficulties with identifying certified therapists who had the capacity to accommodate additional patients into their schedule within the required timeframe (i.e., 8 weeks of their fracture). Given the challenges associated with CBT implementation, only one site was able to actively recruit patients. This site screened 86 patients and enrolled 3 patients (3.5%) over a period of three months. Participants were unable to comply with the in-person CBT, with no participants attending an in-person CBT session. Follow-up at one year could not be assessed as the pilot study was stopped early, three months into the study, due to failure to achieve the other three feasibility criteria.

**Conclusion:**

Our pilot trial failed to demonstrate the feasibility of a trial of in-person CBT versus usual care to prevent persistent pain after surgical repair of traumatic long-bone fractures and re-enforces the importance of establishing feasibility before embarking on definitive trials. Protocol modifications to address the identified barriers include the delivery of our intervention as a therapist-guided, remote CBT program.

**Trial registration:**

ClinicalTrials.gov (Identifier NCT03196258); Registered June 22, 2017, https://clinicaltrials.gov/ct2/show/NCT03196258

## Key messages regarding feasibility

1. What uncertainties existed regarding the feasibility?
  Concerns existed around meeting the composite measure of feasibility: the successful implementation of CBT at each clinical site, recruiting 40 patients within 6 months, treatment compliance, and achieving participant follow-up at one year.
2. What are the key feasibility findings?
  Our pilot trial failed to demonstrate the feasibility of a trial of in-person CBT versus usual care to prevent persistent pain after surgical repair of traumatic long-bone fractures.
3. What are the implications of the feasibility findings for the design of the main study?
  The failure of our pilot trial to demonstrate the feasibility of a definitive trial indicates that protocol modifications are needed to address the identified barriers, including the delivery of the intervention as a therapist-guided, remote CBT program.

## Background

Chronic non-cancer pain has been reported to impact approximately 30% of the population in North America, Europe, and Australia [1–5]. Many patients continue to experience persistent pain and disability at one year after undergoing trauma and surgical fracture repair [6, 7].

Among patients with surgically-managed open upper or lower extremity fractures, 65% report moderate to very severe pain and 35% report moderate to extreme pain interference at one year [8].

Furthermore, surgery and trauma are frequently cited as triggering events responsible for the development of chronic pain, with a survey of 5,130 patients in North Britain reporting that 41% attributed their chronic pain to a traumatic event or surgery [9]. A systematic review of 20 observational studies of operatively managed tibial fractures found the mean incidence of persistent post-surgical pain (PPSP) was 47% (range: 10% to 86%) at an average of 24 months after surgery [10]. Although several risk factors for PPSP have been identified, many of these, such as younger age and female gender, are non-modifiable and thus not amendable to direct intervention [11–13]. However, there are emerging data that suggest patients' beliefs and expectations may be associated with clinical outcomes, including persistent pain [9, 14].

The relationship of psychological factors, behaviours, and cognitive processes to the sensation of pain is well-documented. Stress, distress, anxiety, depression, catastrophizing, fear-avoidance behaviors, and poor coping strategies are associated with both acute and chronic pain [6]. These factors can cause alterations along the spinal and supraspinal pain pathways that influence the perception and experience of pain [15]. The Somatic Pre-Occupation and Coping (SPOC) questionnaire was developed to identify unhelpful illness beliefs among orthopedic trauma patients, and high somatic pre-occupation and poor coping (as measured by the SPOC questionnaire) are strongly associated with PPSP, functional limitations, unemployment, and reduced quality of life 1-year after a traumatic fracture repair [16, 17]. This suggests the possibility that trauma patients with unhelpful illness beliefs could be identified early in the treatment process and targeted for concurrent therapy designed to modify such cognitions to improve their prognosis.

There is a substantial and growing body of orthopaedic literature documenting the importance of psychological factors in predicting recovery from surgery [14, 18]. In a systematic review of the effect of perioperative psychotherapy on persistent pain and physical impairment following surgery, there was a mean reduction in pain of 1.06 cm (95% CI 1.56, 0.55) on the 10cm visual analogue scale for pain when eight randomized controlled trials exploring the effect of active psychotherapy (cognitive behavioral therapy [CBT] with or without relaxation therapy) on the severity of PPSP were pooled [18]. Additionally, when seven randomized controlled trials that explored the effect of active perioperative psychotherapy on long-term physical impairment after surgery were pooled, there was a mean reduction in impairment of 9.87% (95% CI 13.42, 6.32) on the 0-100% Oswestry Disability Index [18]. Collectively, these findings suggest that modifying patient beliefs and thought patterns through CBT may be an effective strategy to improve outcomes among patients who have suffered trauma and require surgery.

The purpose of this pilot study was to assess the feasibility of a randomized controlled trial comparing in-person CBT to usual care alone in operatively managed extremity fractures at high risk of poor outcomes due to unhelpful illness beliefs. The primary outcome planned for the definitive trial was PPSP, with secondary outcomes of health-related quality of life and functional outcomes as demonstrated by return to work and leisure activities.

## Methods

### Study design

We planned for a pilot randomized controlled trial of 40 patients at 4 clinical sites with operatively managed extremity fractures who screen positive for unhelpful illness beliefs (SPOC scores ≥48). The purpose of this pilot trial was to evaluate the feasibility of a larger definitive trial comparing the effectiveness of in-person CBT versus usual care on PPSP, health-related quality of life, and function. The trial was approved by the Hamilton Integrated Research Ethics Board (#3257) and by all participating clinical sites’ research ethics boards/institutional review boards.

### Eligibility criteria

We used broad eligibility criteria to increase the generalizability of our trial: (1) adult men or women aged 18 years and older, (2) screen positive for unhelpful illness beliefs (SPOC scores ≥48), (3) extremity fracture requiring surgery, (4) cognitive ability and language skills to participate in the CBT intervention, and (5) provision of informed consent. The exclusion criteria were: (1) anticipated problems with the patient complying with the study protocol (e.g., impending incarceration), or (2) anticipated problems with maintaining follow-up (e.g., no fixed address).

### Participating sites

Four clinical sites in the United States and Canada participated in our pilot study: 1) R Adams Cowley Shock Trauma Center, Maryland, USA; 2) University Hospital, London, Canada; 3) Royal Columbian Hospital, New Westminster, Canada; and 4) Hamilton Health Sciences – General Site, Hamilton, Canada.

### Screening, enrollment, and randomization

Patients aged 18 or older with an extremity fracture requiring surgery were screened for eligibility. Reasons for ineligibility were documented in the study electronic data capture system. Potentially eligible patients were approached to participate in the trial between 4 and 8 weeks after their injury. This timeframe was chosen since fracture patients typically attend a follow-up visit with their surgeon approximately 6 weeks post-fracture. By this time, acute post-operative pain is typically decreasing, patients are weightbearing on their injured extremity and are starting to return to normal daily activities. Once patients had provided written informed consent, they were assigned a unique study identification number. The research coordinator at each participating site retained a list with patient identifiers and study identification numbers in a secure location. To ensure concealed allocation, eligible patients who provided informed consent were randomized using an online randomization service (www.randomize.net). Due to the nature of the intervention, it was not feasible to blind participants or CBT therapists to treatment allocation.

### Interventions

Consenting participants received either individualized CBT in addition to usual care, or usual care alone (control). The CBT intervention consisted of six, weekly one-hour sessions focused on addressing maladaptive beliefs related to pain and recovery as well as teaching skills to enhance coping and management of pain symptoms. The CBT sessions were developed specifically for this trial and the manual used was based on the CBT for chronic pain protocols developed by Otis (2007) and Thorn (2004) [19, 20]. The intervention was tailored for the treatment of post-fracture patients with acute pain and the specific focus of CBT sessions was informed by individual participant’s responses on the SPOC questionnaire.

The intervention followed a session-by-session protocol that included the following components: (1) emotional processing of the experience of pain and introduction to the cognitive-behavioural model, (2) introduction to the biopsychosocial model of pain, (3) cognitive strategies, (4) behavioural strategies, (5) mindfulness and acceptance, (6) optimizing functioning, and (7) preparing and managing for the future. Between-session homework exercises focused on practicing and applying the skills learned during sessions, as incorporating these skills into daily life is a key component of CBT. Each session included a review of material from the previous session, review of between-session practice, introduction of new skills/material, and establishment of new between-session homework. Psychotherapy was provided by experienced therapists and guided by a CBT manual developed for this study. The control group received usual care alone, as per local clinical practices at each site.

### Outcomes

The primary outcome of the pilot study was a composite measure of feasibility, including: (1) implementation of the CBT intervention at all four clinical sites, (2) rate of recruitment (number of participants recruited over a 6-month period), (3) treatment compliance, and (4) follow-up (proportion of participants followed at one year).

The planned primary outcome for the definitive trial was PPSP at one year after fracture measured using the Brief Pain Inventory (BPI) Short Form. Secondary outcomes included the short form-12 (SF-12) physical component summary (PCS) score, the EuroQuol-5D (EQ-5D), and functional outcomes (defined by participants’ return to work, household activities, leisure activities, and when they achieved 80% of their pre-injury level of function). Outcomes were to be assessed at 3 months, 6 months, 9 months, and 12 months post-fracture at in-person clinic visits or by telephone.

### Feasibility analysis

The primary outcome of feasibility was a composite of the four *a priori* feasibility criteria defined above. Success for each was defined as: 1) successful implementation of CBT at each site, 2) 40 patients recruited within 6 months, 3) treatment compliance in a minimum 36 of 40 participants (90%), and 4) 32 of 40 participants (80%) achieving follow-up at one year. A definitive trial of in-person CBT versus usual care alone would be considered feasible if all four of the feasibility criteria were met.

### Sample size

Since the feasibility objectives in our pilot study did not lend themselves to traditional quantitative sample size calculations, we selected a sample size of 40 patients and an enrollment period of 6 months. This approach allowed us to assess the feasibility of successfully implementing a definitive trial.

### Statistical analysis

Descriptive statistics were used to analyze the baseline characteristics reported by treatment groups as count (percent) for categorical variables and mean (and standard deviation) or median (and interquartile range) for continuous variables, as appropriate. Descriptive statistics were also used to summarize feasibility outcomes.

## Results

### Feasibility criteria 1: successful administration of CBT at each site

Of the four sites initially included in the pilot study, CBT was successfully implemented at only two of the four (50%) sites. The other two clinical sites were unable to implement in-person CBT due to lack of therapist availability. To further evaluate this criterion and to plan for the definitive trial, 30 additional clinical sites, in addition to the four originally proposed, were also approached regarding participation. Many of these clinical sites also experienced similar difficulties in finding at least two qualified and licensed CBT therapists with the capacity to accommodate an increased caseload, as well as ensuring that the qualified therapists would be able to schedule participants within 8 weeks of their fracture (as per the protocol). Overall, the criterion of successful implementation of CBT at each clinical site was not met.

### Feasibility criteria 2: participant enrollment

Of the two clinical sites that successfully implemented CBT, only one clinical site enrolled patients into the trial. In the first three months of recruitment at the Hamilton Health Sciences – General Site, 86 potential trial participants were screened for inclusion. Of these 86, only three participants (3.5%) were enrolled in the study, while 83 patients (96.5%) failed to meet the eligibility criteria (Table 1). The primary reasons for exclusion were related to screening for unhelpful illness beliefs, with 24 (28%) patients not meeting the threshold for unhelpful illness beliefs on the SPOC questionnaire, and 8 (10%) patients declining to complete the screening questionnaire for unhelpful illness beliefs. At the final screening session in which the CBT eligibility questions were asked, 19 patients (22%) were excluded for one of the following reasons: 1) 7 patients (8%) anticipated problems attending CBT sessions, 2) 6 patients (7%) had an active substance use disorder that, in the judgement of the treating surgeon, would interfere in the patient’s ability to partake in the CBT and/or the study, 3) 3 patients (4%) were unable to start the CBT at approximately 8 weeks post-fracture, and 4) 3 patients (4%) were already participating in or planning to start other psychological treatments (including CBT) within the duration of the study (12 months). Additionally, 13 (15%) patients were not screened at approximately 6 weeks post-fracture as required by the trial protocol.

**Table 1:** Screening and enrollment

**Reasons for Exclusion**	**Number of Patients** **n (%)** ***N*** **=86 patients screened in total**
**Initial Screen**	***N*** **=86 screened**
Excluded, reasons below	40 (47%)
Patient was not screened at approximately 6 weeks post-fracture	13 (15%)
Patient has a fragility fracture (a fall from a standing height or less, that results in a fracture)	9 (11%)
Patient is unwilling to complete the SPOC questionnaire	8 (9%)
Patient is unwilling to discuss the study	5 (6%)
Patient does not have the cognitive ability and language skills to participate in CBT interventions	3 (4%)
Patient is facing current or impending incarceration	1 (1%)
Patient did not receive internal fixation within 6 weeks of injury	1 (1%)
**Secondary Screen**	***N*** **=46 screened**
Patient does not meet the threshold for unhelpful illness beliefs on the SPOC questionnaire	24 (28%)
**Final Screen**	***N*** **=22 screened**
Excluded, reasons below	19 (22%)
Patient anticipates problems attending CBT sessions	7 (8%)
Patient has an active substance use disorder that, in the judgement of the treating surgeon, would interfere in the patient’s ability to partake in the CBT and/or the study	6 (7%)
Patient is unable to start the CBT at approximately 8 weeks post-fracture	3 (4%)
Patient is already participating in or planning to start other psychological treatments (including CBT) within the duration of the study (12 months)	3 (4%)
**Eligible after Screening**	***N*** **=3 screened**
Enrolled	3 (4%)

### Feasibility criteria 3: treatment compliance

Of the three participants enrolled, one was randomized to CBT, but they were unable to schedule an in-person CBT therapy session as the CBT therapist did not have any appointments available within 8 weeks of the participant's fracture.

### Feasibility criteria 4: one year follow-up

The pilot study was terminated at 3 months due to failure to meet the prior 3 feasibility criteria, and therefore, we were unable to assess the feasibility criterion of follow-up at 1 year.

## Discussion

The primary finding of this pilot study of in-person CBT for patients with operatively managed extremity fractures was that three feasibility criteria were not met. Delivery of in-person CBT was particularly problematic, as a result of challenges with identifying at least two qualified CBT therapists that could accommodate fracture patients within 8 weeks of their injury. Additionally, participants with fractures experienced difficulties attending these appointments due to the lack of available transportation and mobility issues associated with their injuries. To further explore the challenges faced at our pilot study clinical sites, we vetted the protocol by including 30 additional clinical sites in the United States and Canada. Most of the sites faced similar challenges and were unable to locate at least two qualified therapists who had the capacity to provide rapid access to in-person CBT for trial participants.

Additionally, we were unable to meet the pre-determined enrollment threshold of 40 patients in 6 months. Specifically, one clinical site was initiated and was able to enroll three participants over three months. Given the logistical challenges faced with the in-person CBT and low enrollment, we decided to terminate the pilot study after 3 months. The screening of 86 participants at the one initiated clinical site provided insight into the eligibility criteria for the trial and will be helpful for informing the design of future CBT trials in orthopaedic fracture patients. Specifically, the one initiated clinical site screened 86 patients over 3 months, and of these, only three participants (3.5%) were enrolled in the study. The primary reasons for exclusion were related to screening for unhelpful illness beliefs as many patients did not reach the SPOC score threshold. We discussed the necessity of using unhelpful illness beliefs for screening purposes with our clinical experts (e.g., psychologists, orthopaedic surgeons). Our clinical experts concluded that all fracture patients are at risk of PPSP and poor outcome, and therefore, it is unnecessary to exclude patients who screen low on having unhelpful illness beliefs. They suggested that we avoid using SPOC scores as an eligibility criterion, but stratify our randomization and analysis by the severity of unhelpful illness beliefs at enrollment.

Nineteen of 22 patients were ineligible to attend in-person CBT for various reasons, including the following: 1) patient anticipates problems attending CBT sessions, 2) patient has an active substance use disorder that, in the judgement of the treating surgeon, would interfere in the patient’s ability to partake in the CBT and/or the study, 3) patient is unable to start the CBT at approximately 8 weeks post-fracture, and 4) patient is already participating in or planning to start other psychological treatments (including CBT) within the duration of the study (12 months). Of the 22 patients who made it through the primary and secondary screening, only three were eligible following the final screening. The ability to provide CBT remotely, or use other remote pain treatment options [21, 22], would address several of these barriers.

In addition, 13 (15.1%) patients were not screened at approximately 6 weeks post-fracture as required by the trial protocol. Widening the enrollment window may also increase enrollment and permit additional patients at risk of PPSP to be enrolled.

The pilot study was conducted in 2018, before the global COVID pandemic and at a time when health care and therapy sessions were typically conducted in an in-person setting. While technological advancement has been a driving force behind the development of alternate delivery models for healthcare, the global COVID pandemic also led to widely implemented virtual care models [23, 24]. Although access to technology may be a considerable barrier to some patients, in a systematic review and meta-analysis of 1,418 participants from 20 studies comparing guided internet-delivered CBT to face-to-face CBT for psychiatric and somatic conditions, investigators found that internet-delivered CBT and face-to-face treatment produced equivalent overall effects [25].

Our pilot study revealed that in-person CBT for a trial of orthopedic trauma patients was not feasible and alternatives, such as remote CBT or app-based CBT, should be considered. Second, screening for unhelpful illness beliefs excluded many fracture patients that may benefit from CBT. Third, lengthening the screening window would allow for more fracture patients who are at risk of developing long-term pain to be included, as well as provide more time for those who are not yet weight-bearing and experiencing pain at the 6-week post-fracture mark to start regaining mobility.

## Conclusions

Our pilot study revealed several challenges associated with the implementation of in-person CBT for fracture patients, which ultimately led to the inability of the study to meet its pre-determined feasibility criteria. The pilot study did provide important insights to re-designing the trial protocol. The use of internet-based or virtual CBT interventions may improve access for trial participants, and expanding eligibility criteria should improve recruitment. Our pilot study also demonstrated the importance of feasibility studies prior to embarking on definitive trials.

## Data Availability

The datasets used and/or analysed during the current study are available from the corresponding author on reasonable request.

## Abbreviations

BPI: Brief Pain Inventory
CBT: Cognitive behavioural therapy
EQ-5D: EuroQuol-5D
PCS: Physical Component Summary
PPSP: Persistent Post-Surgical Pain
SF-12: Short Form-12
SPOC: Somatic Pre-Occupation and Coping
